# Iterative Monitoring of Temperatures in Confinement for Early Screening of SARS-CoV-2 Infections

**DOI:** 10.3389/fmed.2020.564377

**Published:** 2020-12-04

**Authors:** Shu Yuan, Si-Cong Jiang, Zi-Lin Li

**Affiliations:** ^1^College of Resources, Sichuan Agricultural University, Chengdu, China; ^2^Chengdu KangHong Pharmaceutical Group Comp. Ltd., Chengdu, China; ^3^Department of Cardiovascular Surgery, Xijing Hospital, Medical University of the Air Force, Xi'an, China

**Keywords:** SARS-CoV-2, asymptomatic infection, body temperature, digital tracing, older patients

Since the outbreak of the novel SARS-like coronavirus (SARS-CoV-2), more than 50,000,000 cases have been reported globally. The proportion of infected people with mild or no symptoms may be as high as 59% (1). However, they may still be contagious, as throat swabs detected high levels of the virus early in their illness, as was the case with symptomatic patients (2–8). It is worth noting that the peak viral load of SARS-CoV-2 was more than 1,000 times higher than SARS-CoV-1, and active SARS-CoV-2 replication in upper respiratory tract tissues has been found, whereas SARS-CoV-1 is not thought to replicate at this site (7). Some convenient and efficient large-scale-screening methods specific to mild/asymptomatic patients urgently need to be developed. Ferretti et al. (9) explored the possibility of protecting the population using questionnaires vs. algorithmic instantaneous coronavirus disease 2019 (COVID-19) contact tracing assisted by a mobile phone application. This tracing method may be very useful for identifying infected people (10), however some convenient and pre-diagnostic methods still need to be developed urgently, since people without symptoms do not always take the viral nucleic acid test, even if they have been in close contact with an infected individual.

As COVID-19 detection technology develops, national nucleic acid testing has become feasible in some countries. However, in other countries lacking diagnostic facilities, only symptomatic patients are subjected to the SARS-CoV-2 test. A high body temperature (>37.3°C) is still the most distinguishable diagnostic criteria for SARS-CoV-2 infection.

A previous report using admission data of 41 patients in Wuhan, China, from Dec 16, 2019, to Jan 2, 2020 indicated that the most common symptom at onset of illness was fever, with a ratio of 98% (11). However, a later study with 99 patients in Wuhan from Jan 1 to Jan 20, 2020 demonstrated that 83% of the patients had a clinical manifestation of fever (12). Then a nation-wide clinical study of 1,099 cases from Dec 11, 2019 to Jan 29, 2020 in China found that fever was present in only 43.8% of the patients on admission but developed in 88.7% during hospitalization (13). Similarly, a study with a cohort of 366 patients with laboratory-confirmed COVID-19 in Sichuan, China, from January 2020 to February 2020 indicated that 69.3% of the mild patients and 79.1% of the severe patients showed body temperatures <37.3°C on admission (14). These reports suggested that the virus has converted its infection strategy to adapt to the human body or that more asymptomatic patients (without fever) had been screened out as time went by. Considering that over 70% of COVID patients did not have fever on admission, the current definition of a viral fever of ≥37.3°C cannot discriminate all SARS-CoV-2 cases.

However, normal human body temperature has lowered (from 37°C to 36.5–36.7°C) over the past two centuries worldwide (15–17). The body temperature of older individuals is even lower, with a basal body temperature of 0.23–0.56 degrees less than young individuals (18, 19). A rise of 1.0°C in some older infected individuals would not be defined as a fever case, if their basal body temperature was <36.3°C, which would result in missed diagnosis. Nevertheless, the older the infected individuals are, the more likely they are to develop severe acute respiratory distress syndrome (ARDS) and die (14, 20, 21). The presence of coexisting medical conditions was significantly higher in older patients compared with younger patients (55.15 vs. 21.93%), including hypertension, diabetes, heart disease, and chronic obstructive pulmonary disease. Thus, significantly higher rates of severe clinical type (16.18 vs. 5.98%), critical clinical type (8.82 vs. 0.77%), and shortness of breath (12.50 vs. 3.07%) were observed in older patients compared with younger patients (20). Accurate and early diagnosis of older people may be greatly helpful in reducing COVID-19 mortality.

## Literature Search and Study Selection

To further elucidate whether body temperature can be used as a pre-diagnostic indicator specific to mild/asymptomatic patients, we conducted a literature search of peer-reviewed publications in electronic databases from their inception to November 13, 2020. The databases used in the search procedure were PubMed, Embase, ISI Web of Science, and medRxiv. The following two key terms were employed for the literature search: “body temperature” and “SARS-CoV-2 or COVID-19.” Through these searches, we obtained a total of 175 results in PubMed and 1,735 results in medRxiv, irrespective of the language, date of publication, and nationality, race, age, or gender of the participants. Two authors (SCJ and SY) independently screened the titles and abstracts to remove the ineligible studies. Disagreements were resolved by discussion. We retrieved the full text of the potentially eligible studies and examined full-text reports for further evaluation. In cases where there were multiple reports for the same study, we used the last published report. During the subsequent full-text screening, articles without the average (median) body temperature data from both mild and severe patients were excluded. Finally, only four reports met the criteria.

## A Rise of 0.5°C Would be a Diagnostic Criteria for Most SARS-CoV-2 Infections

Then the above four reports were analyzed further. A nation-wide clinical study of 1,099 cases from Dec 11, 2019 to Jan 29, 2020 in China found that fever (≥37.5°C) was present in only 43.8% of the patients on admission but developed in 88.7% during hospitalization (13). The average body temperature of the patients on admission was 37.3°C, and the average maximum temperature during hospitalization was 38.3°C (13), which were 0.5°C and 1.5°C higher, respectively, than the normal body temperature of 36.8°C (19). Interestingly, there is no significant difference in the median temperature on admission (37.3 and 37.4°C for non-severe patients and severe patients respectively), although the proportion of the severe patients with body temperatures >38.0°C (21.6% of patients had body temperatures of 38.1–39.0°C and 4.7% of patients had body temperatures >39.0°C) was higher than that of the mild patients with temperatures >38.0°C (17.6% of patients had body temperatures of 38.1–39.0°C and 3.3% of patients had body temperatures >39.0°C) (13).

Clinical features of 5,279 patients with SARS-CoV-2 infections in New York City, USA, similarly showed that the average temperatures at presentation for non-hospitalized patients and hospitalized patients were 37.3 and 37.5°C, respectively (22). The proportions of temperatures ≥38°C at presentation were 5.0 and 33.5% for non-hospitalized patients and hospitalized patients respectively (22). Similar body temperatures on admission have been reported for adult inpatients from Korea (37.04 and 37.81°C for 198 cured patients and 13 transferred severe patients, respectively) (23). Slightly higher median temperatures have also been reported in 36 children patients on admission in Zhejiang province, China (37.6 and 38.0°C for mild cases and moderate cases, respectively) (24).

Thus, body temperature is a good indicator for viral infection (either symptomatic or asymptomatic). A rise of at least 0.5°C may be a diagnostic criteria. However, as mentioned above, many patients' basal body temperatures are below 36.8°C (especially in older patients), and a rise of 0.5°C would not be defined as a fever case, resulting in missed diagnosis.

## The Method of Iterative Monitoring of Body Temperatures

**Figure 1:**
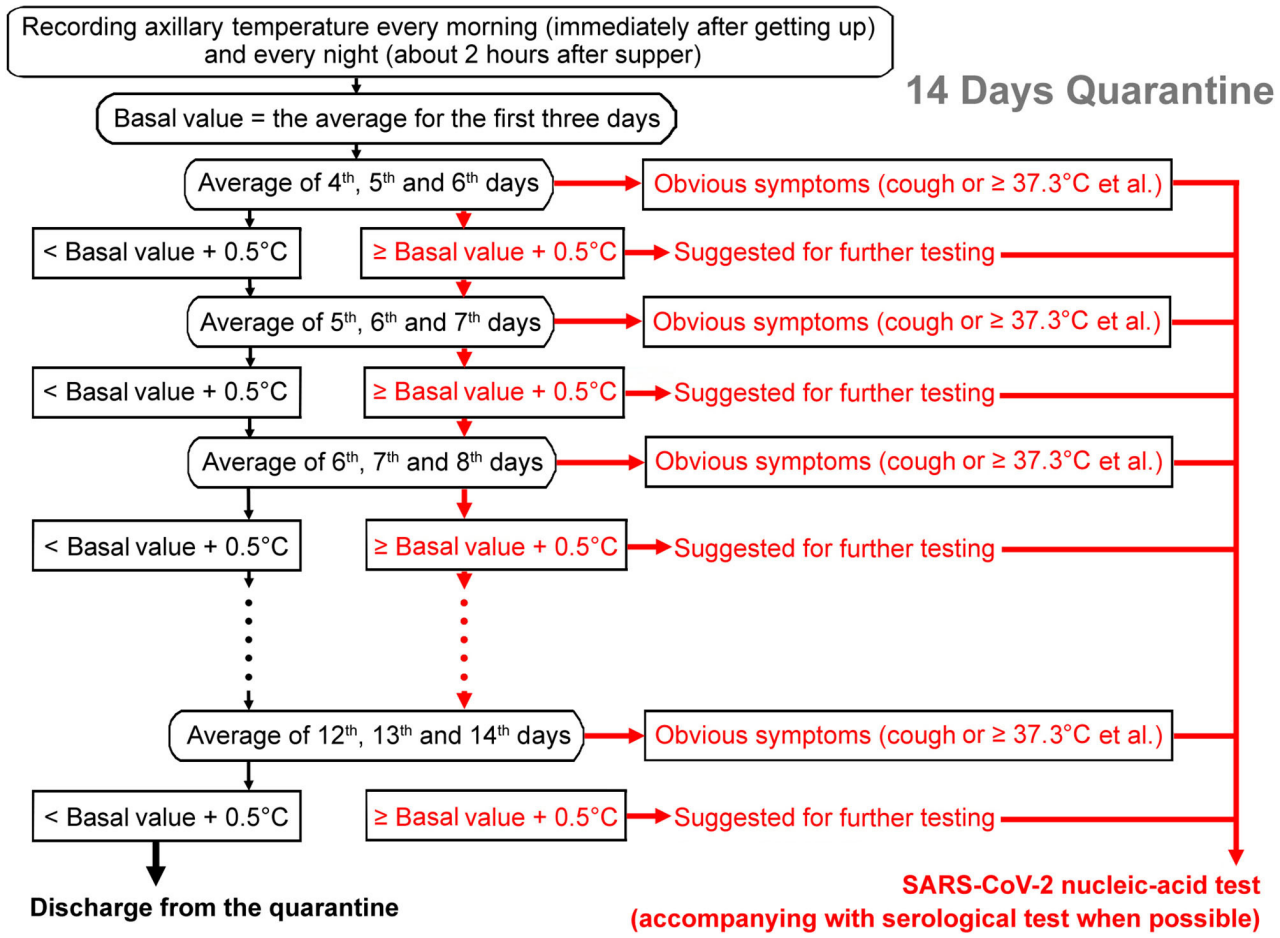
Flow chart of the iterative temperature monitoring method to find possible infections of SARS-CoV-2. The average temperature of the first 3 days of the quarantine period would be taken as the basal value. Then if the average temperature of the next 3 days is more than 0.5°C higher than the reference value, it would be suggested to do a SARS-CoV-2 infection test (pink arrows). If the temperature continued to rise in the following 2 days or apparent symptoms (for example, fever or cough.) developed, it would be strongly suggested to carry out a SARS-CoV-2 infection test (red arrows). If the temperature is not higher than the reference value by more than 0.5°C, then the recording would continue until the end of the 14th day of the quarantine period (black arrows). In addition to a nucleic-acid test, adjunct of serological test is recommended when possible.

How to accurately detect the 0.5°C rise in body temperature poses a big challenge to clinicians. An individual's body temperature can change significantly within a day (<1.0°C), influenced by diet, exercise state, mental factors, and so on (25). A rise of 0.5°C could not be discriminated accurately. In order to reflect the changing trend more accurately, we propose a method of iterative monitoring of body temperatures: recording axillary temperatures of close contacts every morning (immediately after getting up) and every night (about 2 h after supper). The average temperature of the first 3 days of the quarantine period (six measurements) should be taken as the basal value. Then if the average temperature of the next 3 days (the 4th, 5th, and 6th days) could be more than 0.5°C higher than the reference value, it would be suggested to do the SARS-CoV-2 infection test. If the temperature continued to rise in the following 2 days (both the average temperature of 5th, 6th, and 7th days ≥ the basic value + 0.5°C and the average temperature of 6th, 7th, and 8th days ≥ the basic value + 0.5°C), it would be strongly suggested to carry out the SARS-CoV-2 infection test. If it was not more than 0.5°C higher than the reference value, then the recording would continue until the end of the 14th day of the quarantine period (see the flow chart in Figure 1). However, the positive detection rate of a single nucleic-acid test is only 30–50% (26, 27). With the rapid advances in SARS-CoV-2 IgM-IgG antibody tests, adjuncts of serological tests would improve the accuracy in COVID-19 diagnosis. Thus, combined detection of SARS-CoV-2-specific antibodies and nucleic acid has been recommended when possible (26, 27).

This temperature monitoring method could be performed at home without any sophisticated equipment and therefore satisfies the need for large-scale screening, especially in countries lacking diagnostic facilities. The method would also build up an individual temperature reference and therefore satisfies the need for personalized diagnoses. Considering that not all patients could accurately calculate the average temperature by themselves, the everyday temperature recording may be uploaded to an online phone app, as Ferretti et al. (9) proposed, or the calculation could be performed by a computer program (28), and then the CDC would arrange the virus tests for the suspected patients.

## Conclusions

Body temperature is a good indicator for SARS-CoV-2 infection. Evidence profiles of the two reports with 6378 patients showed that the average body temperature of the patients on admission was 37.3°C, which was 0.5°C higher than the normal body temperature of 36.8°C. However, many patients' basal body temperatures are below 36.8°C (especially in older patients with ages ≥60), and a rise of 0.5°C would not be defined as a fever symptom. The current definition of viral fever of ≥37.3°C cannot discriminate all SARS-CoV-2 infections. Here we propose that if the average temperature of 3 days is more than 0.5°C higher than the reference value, it would indicate an infection. If the temperature continued to rise in the following 2 days, it would be strongly suggested to carry out a SARS-CoV-2 infection test. It is now clear that many individuals who are infected (and even those with mild symptoms) do not exhibit fever. Thus, our approach could not discriminate all SARS-CoV-2 infections. However, this method may be helpful to quickly identify contagious asymptomatic patients and older infected individuals with higher risks of death.

## Author Contributions

SY conceptualized the analysis and wrote the original draft. S-CJ and Z-LL reviewed and edited the manuscript. All authors have read and agreed to the published version of the manuscript.

## Conflict of Interest

S-CJ was employed by the Chengdu KangHong Pharmaceutical Group Comp. Ltd. The remaining authors declare that the research was conducted in the absence of any commercial or financial relationships that could be construed as a potential conflict of interest.
